# Annotated Whole-Genome Shotgun Sequence of *Mycobacterium tuberculosis* MTBR2/09 Isolated from a Sputum Sample in Malaysia

**DOI:** 10.1128/genomeA.00515-16

**Published:** 2016-06-23

**Authors:** Rahizan Issa, Valentinus H. Seradja, Mohd Khairul Hafizi Abdullah, Hatijah Abdul

**Affiliations:** Bacteriology Unit, Infectious Disease Research Centre, Institute for Medical Research, Kuala Lumpur, Malaysia

## Abstract

*Mycobacterium tuberculosis* MTBR2/09 was isolated from a sputum sample from a male patient in Malaysia. This is a report of an annotated genome sequence of *M. tuberculosis* MTBR2/09.

## GENOME ANNOUNCEMENT

The previous success of tuberculosis (TB) control in Malaysia is a perplexity. Encounters of TB control can be seen in locations of Malaysia Borneo (1). It was since the early 2000s that the case notification rate for Sabah has been sustained at higher levels of 144 to 217 per 100,000 population (2). In this report, a clinical isolate of *M. tuberculosis* MTBR2/09 from a male patient was subjected to whole-genome shotgun sequencing.

The total genomic DNA of *M. tuberculosis* MTBR2/09 was extracted using QIAxtractor and reagent packs (Qiagen, Düsseldorf, Germany), according to the manufacturer’s instruction. DNA concentrations were measured using a NanoVue Plus (GE Healthcare, Buckinghamshire, United Kingdom) spectrophotometer. The Illumina MiSeq platform was used for the whole-genome shotgun sequencing.

This resulted in 4,303,275 filtered reads, with an average read length of 116.0 bp and approximately 111-fold coverage. The filtered reads were *de novo* assembled with CLC Genomics Workbench version 6.0.1, generating 192 contigs (*N*_50_, 60,366 bp). The genome contains a total of 4,338,492 bp, with a G+C content of 65.58%.

The NCBI Prokaryotic Genome Annotation Pipeline was performed. The genome was identified as having 4,145 genes, 3,946 coding sequences (CDSs), and 150 pseudogenes. Three types of rRNA (5S, 16S, and 23S) were annotated, and each had one rRNA. One clustered regularly interspaced short palindromic repeat (CRISPR) array, one noncoding RNA (ncRNA), and 45 tRNAs were annotated in this genome.

The SpolPred software was used to determine the spoligotype of *M. tuberculosis* MTBR2/09. It was determined to be 000000000003731, belonging to East Asian (Beijing) lineage.

### Nucleotide sequence accession number.

This whole-genome shotgun project has been deposited in DDBJ/ENA/GenBank under the accession no. LATO00000000. The version described in this paper is the first version.
